# 1320. Pneumococcal Meningitis Before and During Covid – 19 Pandemic in Uruguay, South America (2017 – 2022).

**DOI:** 10.1093/ofid/ofad500.1159

**Published:** 2023-11-27

**Authors:** Marcos Delfino, Ana P Méndez, Monica Pujadas Ferrer, María Catalina Pírez

**Affiliations:** Faculty of Medicin, University of the Republic, Montevideo, Montevideo, Uruguay; Faculty of Medicine. University of the Republic. Uruguay, Montevideo, Montevideo, Uruguay; Faculty of Medicine University of the Republic Uruguay, Montevideo, Montevideo, Uruguay; Faculty of Medicin, University of the Republic, Montevideo, Montevideo, Uruguay

## Abstract

**Background:**

In Uruguay, universal vaccination of children with 7/13 valent pneumococcal conjugate vaccines was introduced between 2008 and 2010 (2 + 1 schedule), achieving nationwide coverage of 95%. This has caused a significant decrease in pneumococcal meningitis (PM).

The Covid-19 pandemic led to worldwide quarantines and isolation during 2020 and 2021. This resulted in a significant decrease in other infections. With the mass vaccination campaign worldwide, in 2022 we could return to a society life like before 2020. However, in 2022 there was an unusually high circulation of infections caused by other viruses and bacteria.

The objective of this study is to compare the number of cases of PM before and during the early and late pandemic of Covid-19 in the general population in Uruguay.

**Methods:**

This descriptive, retrospective study includes all cases reported to the Department of Health Surveillance of the Ministry of Public Health with PM between January 1, 2017, and December 31, 2022. It also includes all SARS CoV 2 infections informed in Uruguay. PM variables are cases per year, sex, age (≤ 15 years and > 15 years), and deaths by period. The PM average rates of cases per 100,000 inhabitants are described in three periods: Period 1 or pre – pandemic (P1, 2017 to 2019); Period 2 or early – pandemic (P2, 2020 and 2021), and Period 3 or late – pandemic (P3, 2022). Statistical analysis were established based on frequency distribution and statistical significance tests as appropriate, considering a value of *p* equal to or less than 0.05 as statistically significant. The institutional Ethics Committee approved the research.

**Results:**

The total number of cases PM is 100, 62% are men. The N for P1 is 59, for P2 it is 12 and 29 in P3.

Table 1 shows the main incidence and mortality results by age group in the three periods. Graph 1 shows the evolution of PM in relation to the development of the Covid-19 pandemic in our country. Lethality remains high (24% average for the total period).

**Figure:**
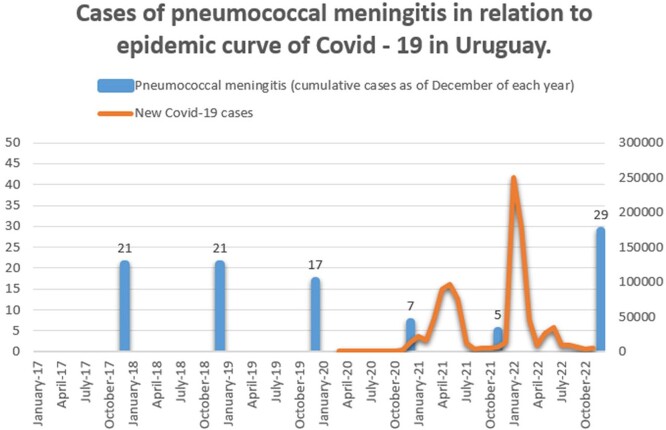

Incidence rates of pneumococcal meninigtis and mortality by period and age.

**Figure:**
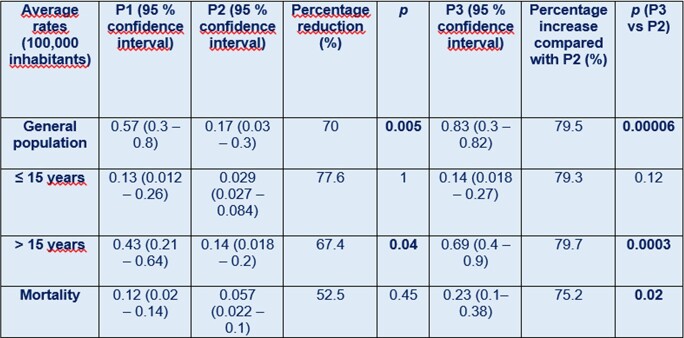

**Conclusion:**

There was statistically significant changes in cases of PM in the general population, in individuals > 15 and mortality. It is very likely that social distancing and the use of facemasks influenced these phenomena. These measures cease after the massive vaccination campaign against Covid-19, which could explain the 2022 increase in PM.

**Disclosures:**

**Marcos Delfino, Pediatrician, Pediatric Infectious Diseases**, Pfizer: Finna **María Catalina Pírez, Pediatrician, Pediatric infectologist, microbiologist Professor of pediatric, Degree V**, Merck, Pfizer: Expert Testimony|Merck, Pfizer: Honoraria

